# QTL mapping for resistance against cereal cyst nematode (*Heterodera avenae* Woll.) in wheat (*Triticum aestivum* L.)

**DOI:** 10.1038/s41598-022-12988-7

**Published:** 2022-06-10

**Authors:** Saksham Pundir, Rajiv Sharma, Deepak Kumar, Vikas Kumar Singh, Deepti Chaturvedi, Rambir Singh Kanwar, Marion S. Röder, Andreas Börner, Martin W. Ganal, Pushpendra Kumar Gupta, Shailendra Sharma, Shiveta Sharma

**Affiliations:** 1 Present Address: Department of Genetics and Plant Breeding, Chaudhary Charan Singh University (CCSU), Meerut, Uttar Pradesh 250 004 India; 2 Department of Botany, Chaudhary Charan Singh University (CCSU), Meerut, Uttar Pradesh 250 004 India; 3 Scotland’s Rural College (SRUC), Peter Wilson Building, West Mains Road, Edinburgh, EH9 3JG UK; 4 Department of Nematology, Chaudhary Charan Singh Haryana Agricultural University (CCSHAU), Hisar, Haryana 125 004 India; 5 Leibniz Institute of Plant Genetics and Crop Plant Research (IPK), Corrensstrasse 3, 06466 Seeland, OT Gatersleben, Germany; 6 Trait Genetics GmbH, Am Schwabeplan 1b, 06466 Seeland, OT Gatersleben, Germany; 7 Murdoch’s Centre for Crop & Food Innovation, Murdoch University, Murdoch, WA 6150 Perth, Australia

**Keywords:** Genetics, Plant sciences

## Abstract

The resistance to cereal cyst nematode (*Heterodera avenae* Woll.) in wheat (*Triticum aestivum* L.) was studied using 114 doubled haploid lines from a novel ITMI mapping population. These lines were screened for nematode infestation in a controlled environment for two years. QTL-mapping analyses were performed across two years (Y1 and Y2) as well as combining two years (CY) data. On the 114 lines that were screened, a total of 2,736 data points (genotype, batch or years, and replication combinations) were acquired. For QTL analysis, 12,093 markers (11,678 SNPs and 415 SSRs markers) were used, after filtering the genotypic data, for the QTL mapping. Composite interval mapping, using Haley-Knott regression (hk) method in R/QTL, was used for QTL analysis. In total, 19 QTLs were detected out of which 13 were novel and six were found to be colocalized or nearby to previously reported *Cre* genes, QTLs or MTAs for *H. avenae* or *H. filipjevi*. Nine QTLs were detected across all three groups (Y1, Y2 and CY) including a significant QTL "*QCcn.ha-2D*" on chromosome 2D that explains 23% of the variance. This QTL colocalized with a previously identified *Cre3* locus. Novel QTL, *QCcn.ha-2A*, detected in the present study could be the possible unreported homeoloci to *QCcn.ha-2D*, *QCcn.ha-2B.1* and *QCcn.ha-2B.2.* Six significant digenic epistatic interactions were also observed. In addition, 26 candidate genes were also identified including genes known for their involvement in PPNs (plant parasitic nematodes) resistance in different plant species. *In-silico* expression of putative candidate genes showed differential expression in roots during specific developmental stages. Results obtained in the present study are useful for wheat breeding to generate resistant genetic resources against *H. avenae.*

## Introduction

Plant parasitic nematodes (PPNs) cause global annual crop yield losses of ~ $173 billion (10%)^1^. Cereal cyst nematodes (CCNs) are one of the most important groups of PPNs attacking cereals^2^. These nematodes are known to cause > 70% significant yield losses in intolerant cultivars of small grain cereals^3^. Three species (*H. avenae*, *H. filipjevi*, and *H. latipons*) of the *H. avenae* group are the most widespread and damaging of the cyst nematode pests that parasitize cereals including wheat, barley, oats, rye, maize and some related crops, wild crop ancestors and wild grasses^4–8^.

PPNs can be controlled by several methods such as chemical control using nematicides, cultural control such as sanitation, flooding, fallowing, crop rotation and biological control by natural predators or pathogens of nematodes. Crop rotation in particular is a very successful and old method to control nematode infection. Although these cultural control methods are quite effective, they cannot be the permanent solutions for growers. In the past, nematicides have been effectively used to control nematodes^7,9^. Smiley et al.^10^ reported that using aldicarb, a nematicide improves the yield of spring wheat. Sharma^11^ observed that Carbofuran and Phorate were more potent and compatible to mitigate the nematode infection as well as endomycorrhizae. However, the use of chemicals, particularly nematicide is not an eco-friendly method therefore their usage is either banned or discouraged in several countries. They were also linked to a polluting environment, which has concerns for humans and other beneficial microorganisms.

Keeping in view the above, there is a need for genetic control in the form of resistance to CCNs in wheat to effectively inhibit or decrease nematode reproduction on this important crop. The use of tolerant and resistant wheat cultivars is the most environmentally safe and often the most effective means to control CCNs like *H. avenae* that deter the use of chemicals.

Till today, 14 CCN resistance genes have been reported which include the following: *Cre1*, *Cre2*, *Cre3*, *Cre4*, *Cre5*, *Cre6*, *Cre7*, *Cre8*, *Cre9*, *CreR*, *CreV*, *CreX*, *CreY* and *CreZ*^12–14^. The majority of these genes are from wild grasses and bread wheat relatives i.e., *Aegilops tauschii* (*Cre3* and *Cre4*)^15,16^, *Ae. ventricosa* (*Cre2*, *Cre5* and *Cre6*)^17–19^, *Ae. triuncialis* (*Cre7*)^20^, *Ae. peregrina* (*CreX* and *CreY*)^21^, *Secale cereale* (*CreR*)^22^ and *Dasypyrum villosum* (*CreV*)^23^*.* Three genes i.e*. Cre1*, *Cre8* and *Cre9* have been reported from the common wheat gene pool. *Cre1* was reported from wheat landrace AUS10894^24,25^. *Cre8* was identified in the Festiguay cultivar^26,27^. *Cre9* was also reported in a wheat cultivar, Madsen, which was originally derived from *Ae. ventricosa* via VPM-1^28^. Another CCN resistance gene analog designated as *CreZ* was isolated from the CCN resistant E-10 near isogenic line (NIL) of wheat^29^. In addition to these, four resistance genes i.e. *Ha1-4* were derived from barley^30,31^.

Recent advances in high throughput genotyping and sequencing technology have made it possible to dissect the whole plant genome to identify genes of interest and their further application^32^. This approach helps to detect single genes or individual nucleotides utilizing the genetic diversity available in natural populations. In order to study the genetics of plant disease resistance and other complex traits, QTL interval mapping is considered as a highly effective approach and helps to identify the locations and determine the effects of the loci governing the trait. The research to date has tended to focus on locating these QTLs rather than understanding the complexity of the traits. In several instances, the complexity of the genetic architecture can be largely attributed to epistatic effects which play a significant role in heterosis, inbreeding depression, adaptation, reproductive isolation and speciation^33^. The precise estimation of epistatic effects of QTL was important for application in marker-assisted selection (MAS). The epistatic interactions analysis can lead to the identification of a QTL that may itself have no effect on a trait, but has a significant effect when interacts epistatically with another QTL^34^. Neglecting epistatic interactions may result in the underestimation of genetic variance and to overestimation of individual QTL effects and also considerable loss of MAS efficiency, especially in later generations^35,36^. Epistasis analysis can assist in assembling a large number of favorable alleles in MAS or introgression^37^.

The main aim of the present study is to identify QTLs governing resistance to *H. avenae* in wheat. We also performed epistatic interactions analysis to detect QTLs, each involved in a significant epistatic effect with another QTL.

## Results

### Phenotypic evaluation of doubled haploid lines (DHs) for *H. avenae* infection

**Figure 1:**
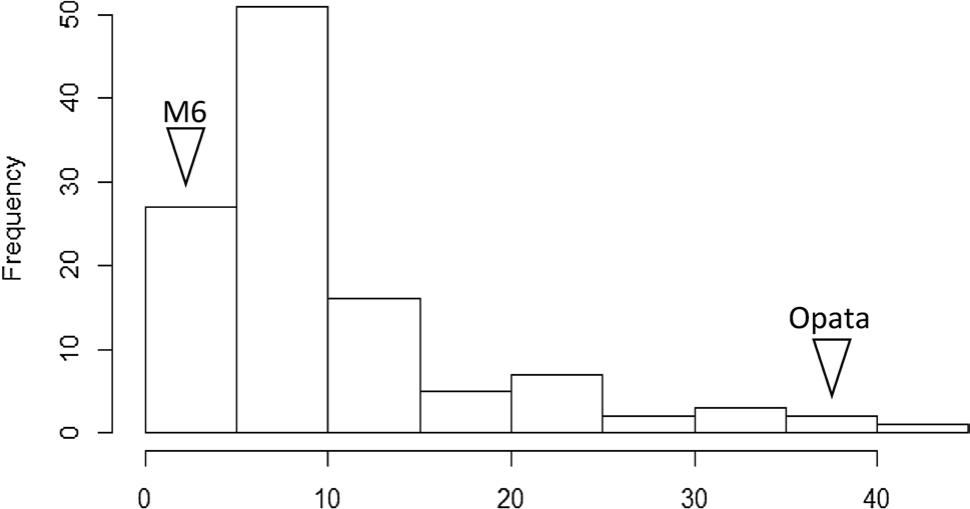
Frequency distribution of doubled haploid (DH) lines for cysts count.

**Figure 2:**
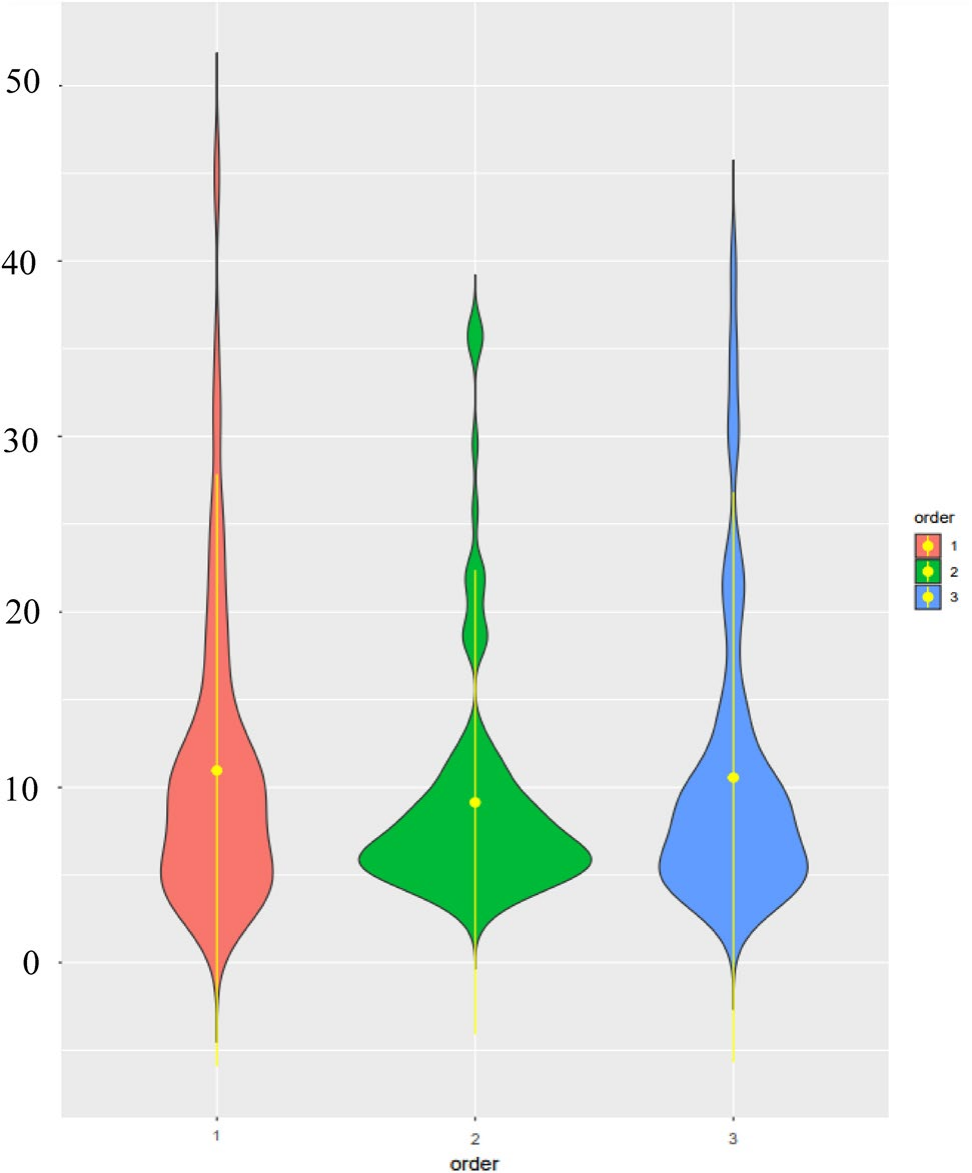
Violin plots showing the distribution of cysts count in Y1 (red), Y2 (green) and combined year data analysis CY (blue). In each case, the vertical solid bar indicates the range of average values, and median is shown as a white circle, depicting the lower, medium and upper quartile.

In total, 114 DHs were screened for *H. avenae* infection for two years. The frequency distribution for the cysts extracted is presented in Fig. 1. The frequency distribution of cyst count displayed skewed distribution towards resistance. Parental genotypes, in terms of the nematode infection in both the experiments displayed expected resistance (W-7984 or M6) and susceptibility (Opata M85) indicating that the screening was quite robust across the experiments (Supplementary Table S1). A wide range of phenotypic variability (mean ± standard deviation value of 10.56 ± 8.09) was observed among the doubled haploid (DH) lines for cysts count that ranged from 2.52 to 40.54 (CV = 0.77) (Fig. 2; Supplementary Tables S1, S2a). There was also evidence of transgressive segregation in the population. Three DH lines displayed CCN counts (< 3.19) that were significantly (*p* < 0.05) lower than the resistant parent and two lines displayed susceptibility higher than the susceptible parent (> 36.40) (*p* < 0.05). These observations indicate the distribution or presence of favorable alleles among both parents. The heritability was estimated to be 0.83 (Supplementary Table S2b).

### QTL analysis

**Figure 3:**
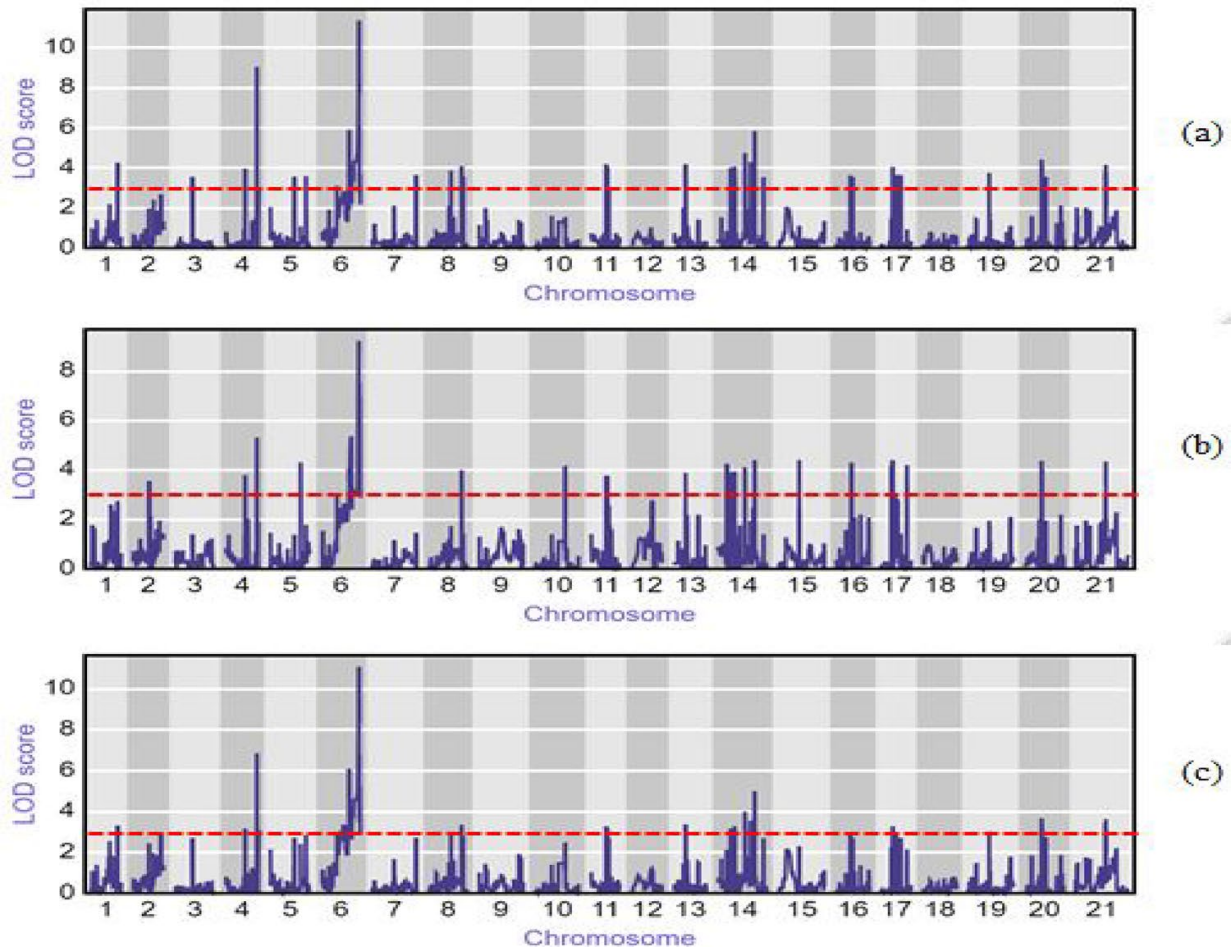
Significant QTL peaks in Y1 (**a**), Y2 (**b**) and CY (**c**) using CIM method in DH lines of ITMI population.

QTL analysis was performed using a high-density linkage map containing > 12 K markers (12,093 markers). Marker distribution across the wheat genome, as well as the sub-genomes, is provided in Supplementary Fig. S1. Recombination breakpoints across the DH lines are displayed in Supplementary Fig. S2. A total of 15 QTLs for year 1 (Y1), 13 for year 2 (Y2) and 11 QTLs for combined data (CY) were detected (LOD value > 3.0) (Fig. 3; Table 1) using CIM. These QTLs were distributed on 18 of the 21 chromosomes (except 3D, 4D and 6D). The LOD values of detected QTLs ranged from 3.02 to 11.37, with additive effects from 0.025 to 3.925 and average phenotypic variance from 0.0007 to 22.84% (Table 1).

**Table 1:** List of QTLs detected for *Heterodera avenae* resistance using composite interval mapping (CIM) in the doubled haploid (DH) population.

S. No	QTLs	Marker	Type	Chr	Pos	LOD	R^2^	Add	Year
1	QCcn.ha-2D*	Excalibur_rep_c67599_2154	SNP	2D	136.40	9.23–11.37	0.2284	3.925	Y1, Y2, CY
2	*QCcn.ha-2A*	Kukri_rep_c68068_95	SNP	2A	113.40	5.30–9.05	0.03971	− 1.628	Y1, Y2, CY
3	QCcn.ha-5B*	IACX6116	SNP	5B	134.40	4.39–5.86	0.04186	− 1.662	Y1, Y2, CY
4	*QCcn.ha-7B*	wsnp_Ex_c26747_35976442	SNP	7B	60.60	3.67–4.42	0.02799	− 1.086	Y1, Y2, CY
5	*QCcn.ha-6B*	BS00045453_51	SNP	6B	42.80	3.26–4.39	7.02E− 06	0.025	Y1, Y2, CY
6	*QCcn.ha-7D*	wsnp_Ex_rep_c68671_67525179	SNP	7D	111.50	3.63–4.34	0.01733	− 1.070	Y1, Y2, CY
7	*QCcn.ha-5A*	wsnp_Ku_c21275_31007309	SNP	5A	41.90	3.37–4.18	0.003085	0.455	Y1, Y2, CY
8	*QCcn.ha-4B*	Tdurum_contig15737_728	SNP	4B	56.40	3.28–4.17	0.01052	− 0.828	Y1, Y2, CY
9	*QCcn.ha-3B*	Tdurum_contig15050_103	SNP	3B	118.30	3.34–4.09	0.001591	0.324	Y1, Y2, CY
10	*QCcn.ha-1A*	Ku_c3523_1959	SNP	1A	99.10	3.31–4.25	0.002364	− 0.393	CY, Y1
11	*QCcn.ha-7A*	wsnp_Ku_c6065_10682531	SNP	7A	79.50	3.02–3.75	0.01782	1.086	CY, Y1
12	QCcn.ha-5D*	wms0174	SSR	5D	75.60	4.41	0.00035	0.125	Y2
13	QCcn.ha-2B.1*	wsnp_RFL_Contig3917_4326857	SNP	2B	109.70	4.25	0.00045	− 0.139	Y2
14	*QCcn.ha-4A*	Kukri_c29142_1046	SNP	4A	111.10	4.11	0.01452	− 0.79	Y2
15	*QCcn.ha-3A*	BS00086051_51	SNP	3A	162.90	3.65	0.00144	− 0.32	Y1
16	*QCcn.ha-6A*	RAC875_c29850_102	SNP	6A	52.20	3.63	0.00015	0.105	Y1
17	QCcn.ha-2B.2*	Tdurum_contig12159_468	SNP	2B	129.40	3.59	0.01943	− 0.31	Y1
18	*QCcn.ha-1D*	wsnp_RFL_Contig2036_1264133	SNP	1D	62.20	3.56	0.000279	− 0.14	Y1
19	QCcn.ha-1B*	Ku_c1932_1583	SNP	1B	59.60	3.54	0.004933	− 0.465	Y2

Chr: Chromosome; Pos: Position; cM): Centimorgan: LOD: Logarithm of the odds; R^2^: Phenotypic Variance (for S. No. 1–11, phenotypic variance of CY and for S. No. 12–19: phenotypic variance of individual year); Add: Additive Effect (for S. No. 1–11, additive effect of CY and for S. No. 12–19 additive effect for of individual year); Y1: Year 1, Y2: Year 2, CY: Combined year; Asterisk indicates QTLs colocalized or nearby to previously reported *Cre* genes, QTLs or MTAs for *H. avenae* or *H. filipjevi.*

A major QTL designated as *QCcn.ha-2D* was detected on chromosome 2D in both years and CY (combined data) (Table 1; Fig. 3; Supplementary Figs. S3, S4, S5, S6). This QTL was linked to marker Excalibur_rep_c67599_2154 with LOD values ranging between 9.23 and 11.37 in the analysis of Y1, Y2 and CY and located at the distal end of chromosome 2D (136.40 centimorgan (cM)). This QTL explained 23% of the phenotypic variance in the combined year analysis with 3.925 additive effects (Table 1). The resistance allele for this QTL was derived from the parent M6.

Another significant QTL, *QCcn.ha-2A* detected on chromosome 2A with LOD values ranged in between 5.30 and 9.05 at 113.40 cM in Y1, Y2 and CY analysis. This QTL was linked to marker Kukri_rep_c68068_95. This QTL explained only 3% of the total phenotypic variance in the combined year analysis with 1.628 additive effects. On chromosome 5B, another significant QTL designated as *QCcn.ha-5B* linked to SNP marker IACX6116 at 134.40 cM was detected across Y1, Y2 and CY. The LOD value for this QTL ranged between 4.39 and 5.86 and explained 4% of phenotypic variance with 1.662 additive effects. The allele for this QTL comes from the susceptible parent, Opata M85. Alleles for the following six minor QTLs: *QCcn.ha-3B*, *QCcn.ha-4B*, *QCcn.ha-5A*, *QCcn.ha-6B*, *QCcn.ha-7B* and *QCcn.ha-7D* also come from Opata M85. The LOD value for these QTLs ranged in between 3.26 and 4.42; the phenotypic variance explained for CY ranged from 0.1 to 2%. The additive effect for these QTLs ranged from 0.025 to 1.086. On chromosome 2B two minor QTLs, namely *QCcn.ha-2B.1* and *QCcn.ha-2B.2* were detected at 109.70 cM and 129.40 cM and linked to SNP markers wsnp_RFL_Contig3917_4326857 and Tdurum_contig12159_468, respectively. The QTL *QCcn.ha-2B.1* was detected in Y1 whereas *QCcn.ha-2B.2* was detected in Y2.

Two QTLs, *QCcn.ha-1A* and *QCcn.ha-7A* were detected only in Y1 and CY on chromosomes 1A and 7A, respectively. The QTL on chromosome 1A is located at 99.10 cM and linked to SNP marker Ku_c3523_1959*.* The LOD value for this QTL ranged between 3.31 and 4.25. Another QTL, *QCcn.ha-7A* is linked to SNP marker wsnp_Ku_c6065_10682531 at position 79.50 cM position; its LOD score value ranged between 3.02 and 3.75. These QTLs explained 0.2% and 1% of total phenotypic variance in CY analysis with 0.393 and 1.086 additive effects, respectively.

Besides the above QTLs, three minor QTLs, *QCcn.ha-1D, QCcn.ha-3A* and *QCcn.ha-6A* were detected in Y1 on chromosome 1D, 3A and 6A, respectively. These QTLs were linked to marker wsnp_RFL_Contig2036_1264133, BS00086051_51 and RAC875_c29850_102 at position 62.20, 162.90 and 52.20 cM. Three minor QTLs were designated as *QCcn.ha-1B*, *QCcn.ha-4A* and *QCcn.ha-5D* on chromosomes 1B, 4A and 5D were detected only in Y2. These QTLs were linked to markers Ku_c1932_1583, Kukri_c29142_1046 and wms0174 at positions 59.60, 111.10 and 75.60 cM (Table 1). Epistatic interaction analysis was performed to check the possible interaction of QTLs with other loci. Summarized information about the main effect QTLs, with involvement in nematode resistance, showing epistatic interaction with other loci is provided in Table 2. Details of all epistatic interactions are given in Supplementary Table S3. For Y1 and Y2, four epistatic interactions (two each) and for CY three epistatic interactions were detected at threshold LOD value > 3.0. Interactions between pairs of QTLs on 4B (57/58 cM), 4D (112 cM), and 6B (63 cM) were common in Y1 and CY.

**Table 2:** Details of epistatic QTL interactions.

Year	Chr 1	Pos 1	Left marker 1	Right marker 1	Chr 2	Pos 2	Left marker 2	Right marker 2	LOD	PVE (%)	Add1	Add2	Add × Add
Y1	4B	57	wms4465	BS00064935_51	4D	112	D_contig24181_338	CAP11_c896_88	4.28	2.07	− 5.4999	3.9676	− 5.2828
	4B	58	Tdurum_contig94032_1688	Kukri_c13401_973	6B	63	Tdurum_contig76997_664	BS00064885_51	3.27	1.53	− 1.0191	0.4055	− 2.5705
Y2	2A	118	BS00021937_51	Kukri_c52608_142	2D	138	wsnp_RFL_Contig2104_1368653	Excalibur_c1451_660	3.17	2.36	− 4.0129	3.3128	− 4.9531
	2D	137	wms0349	RAC875_c30919_311	3D	61	dms0072	D_GBUVHFX02G2BTK_399	3.69	1.02	0.5873	1.9849	2.3166
CY	4B	57	wms4465	BS00064935_51	4D	112	D_contig24181_338	CAP11_c896_88	3.49	2.19	− 4.6946	3.4767	− 4.5798
	4B	58	Tdurum_contig94032_1688	Kukri_c13401_973	6B	63	Tdurum_contig76997_664	BS00064885_51	3.01	1.84	− 0.8286	0.5607	− 2.3508
	7A	78.6	Tdurum_contig60128_346	BS00034689_51	7B	70	wmc0396xxx-a	IAAV7912	3.02	1.7	− 0.1944	− 0.1212	− 2.469

Y: Year (Y1: Year 1; Y2: Year 2; CY: Combined data); Chr: Chromosome; Pos: Position; LOD: Logarithm of the odds; PVE: Phenotypic variance explained; Add: Additive Effect.

### Candidate gene analysis and *in-silico* gene expression

Fifteen QTLs on chromosomes 1A, 1B, 2A, 2B, 2D, 3A, 4B, 5A, 5B, 5D, 6B, 7A, 7B and 7D were linked to putative candidate genes known to be involved in plant-pathogen interactions. We identified a total of 47 putative candidate genes (Supplementary Table S4) for 19 identified QTLs associated with *H. avenae* resistance; only 25 of these candidate genes have a putative role in several disease resistance (Table 3). No hit was found for Kukri_c29142_1046 marker located on chromosome 4A. The maximum numbers of candidate genes (six each) were detected on chromosomes 2D and 3A linked with Excalibur_rep_c67599_2154 and BS00086051_51 markers. According to the Wheat Expression Browser database, the majority of these genes were found to be expressed in wheat tissues including roots (Fig. 6; Supplementary Tables S5, S6). Interestingly, we identified a number of genes showing differential expression in roots. We also selected nine high confidence CGs, with the majority showing more than 3 TPM (transcripts per -million) expressions in wheat roots including genes like homeobox domain, glycosyltransferase family 92 and family 8 and galacturonosyltransferase known for their involvement in PPN resistance.

**Table 3:** Details of disease related candidate gene showing putative associations with *Heterodera avenae* resistance in wheat.

Markers	Marker sequence	Gene ID	Chr	Start	End	Description
Excalibur_rep_c67599_2154	TGTCTATCGTATTAGGTGCTGGATCCAGGGAATAATGTGCTGACAGCTTA[T/G]CATGACTTGCCAGGTTTTGGAAGCATGCTCTGATCGTTTATGAAATATCT	*TraesCS2D02G597900.1*	2D	650,325,169	650,325,279	AT-hook motif nuclear-localized protein
		*TraesCS2D02G598000.1*				Glutathione peroxidase, Thioredoxin-like superfamily
		*TraesCS2D02G598200.1*				C2 domain, Phosphoribosyltransferase C-terminal
Kukri_rep_c68068_95	GATCCTAGATTGACAGGAAGCTTCTCTCTACTTGCTGTTTGTACCTTCTC[A/G]ATGCCCAGCCAGACGGGGCCGACGTCAGCTTCTTTCTACATTCCCGACAC	*TraesCS2A02G567600.1*-*TraesCS2A02G567600.2*	2A	766,400,394	766,400,504	Homeobox domain
		*TraesCS2A02G567700.1*				Peptidase T2, Asparaginase 2
IACX6116	TCCTCGAGCATCAGCTAGTGCAGTAGCATTTGGTTTGGGGCTACTTTCTGGGAAAGGAAA[A/G]CTTGGAGCAGGAAATAATCGTGCCTTCTCTGTTCTGAGCGAGAGTCGTGCAAGTGATAT	*TraesCS5B02G486400.1*	5B	657,711,642	657,711,772	Ubiquitin-like domain superfamily
		*TraesCS5B02G486500.1*				NAD-dependent epimerase/dehydratase
wsnp_Ex_c26747_35976442	AGTGACTGAGCTAAGTTTAGACCAGGCACTATGCTGTTCTGAGTATTCATATCTTTCTTGCATATCTCCTCACCAAGCCATGGCATGGTTCTCTTAAAAA[T/C]ATTATCCATTCCAGAGGTATCATCATCTATCATCCCAGGTTGCCTCGCACACTTTGCGGTGAATAATGGTGTGGGATAGATGAAGAAAGGAGCGATGACA	*TraesCS7B02G138800.1*	7B	174,571,382	174,571,523	S-adenosyl-L-methionine-dependent methyltransferase
BS00045453_51	TTCTTTCAAATGGGTACTGCGGCAGATATTGCAAATAGATGAGTTCTGTA[T/C]TCTCCTAAGGTTCTCATTTGGTATCACCCATGCTCCATGCCAACGATGCC	*TraesCS6B02G129600.1*	6B	126,145,314	126,145,424	Armadillo-type fold, DNA polymerase V/Myb-binding protein 1A
wsnp_Ex_rep_c68671_67525179	TGGAAGCTACTCAGTCAGCAGAATTCCAGAGATGTATGGTGCCACTTTTCCGACAGATTGCCCGCTGTTTAAATAGCTCTCACTTTCAGGTGGCAGAGAG[A/G]GCTCTGTTTCTGTGGAACAACGATCATATTGAGGGTTTGATCAAACAAAACAGCAAGGTGTTACTGCCCATAATCCTTCCTTCATTAGAACGAAATACAA	*TraesCS7D02G310200.1*	7D	393,578,454	393,578,579	Armadillo-like helical, Armadillo-type fold
		*TraesCS7D02G310300.1*				Glycosyltransferase family 92
wsnp_Ku_c21275_31007309	GTCACCAGTACCTTCAACTCCAAATCCAGTTCTGCCACTAACATCACCGCGAGGTTTGACTATGACATCTAGCGTTTCCTCAGCTGCGTCGAATGTGTTG[A/C]CAAGTAGGGGTGTTGGGCCTTCCGCATCAGGCACGCTGCAGTCAGATCCTGATCCAGCACGCTGGATGAATGGCTAACAGGAAGGCTTGGGCTGCACAGT	*TraesCS5A02G365500.1*	5A	565,550,070	565,550,290	P-loop containing nucleoside triphosphate hydrolase
Tdurum_contig15737_728	TCCGGTGTCTACGACGACGACCACTACTTCTGCGATGCCTGTGAGAGTGA[T/C]GTGGTCTGCATCAAGGGTTATTACAATGGCATAGATCAGCAGCTGCTCGA	*TraesCS4B02G130000.1*	4B	169,935,783	169,935,893	Dirigent protein, Jacalin-like lectin domain superfamily
Ku_c3523_1959	CTCGCCATCGCGCCAGAGTTCTTGGTTTTCCCCTCTTCCGCTCGTCAAAC[T/C]TCGCTGCAGAGTGCAGAGTGCCGACCATTCCCCGC	*TraesCS1A02G370800.1-TraesCS1A02G370800.3*	1A	547,963,972	547,964,058	Glycosyl transferase, family 8, Galacturonosyltransferase
		*TraesCS1A02G370900.1*				F-box associated interaction domain, F-box domain, F-box-like domain superfamily
wsnp_Ku_c6065_10682531	ATCTGGGTACTTAACACGCAACTGTAACCGCAAGGACTCAGGACATAGGATCCAATTCATAAGATTGTGTGCACCAGATCAATTGATATGAAG[T/C]ATTTGATTCAACAAATGCATATAGCAGCGGCAGTATATACACCACRGTACCACAGAAGCTAACATGCTGAAAAAGAATTCGATCTTCAGCATGTGACTGT	*TraesCS7A02G156900.1*	7A	109,843,365	109,843,577	Zinc finger, RING-type,Zinc finger, RING/FYVE/PHD-type
wms0174	GGGTTCCTATCTGGTAAATCCCCAACCCTCCTCCGCTACGAGAACTCCTCACCGACGAAGGAATAAAAAAAGGTTTTACGGGCTGCCTATATATGTTTACCGGCTGGGTCATTTTAACAGCATCTGCTCGGACTAAAATTTTCACGTATCTTGTAAATCGGCTGGGATTTTAAGGCCGGTGAGCTTATACGGGGTCTGCTAGAGATGCTCATCGGATCCGGCGCGTTTTAGGGTTCCTATCTGGTAAATCCC	*TraesCS5D02G344400.1*	5D	431,893,813	431,893,837	ATP-binding domain, P-loop containing nucleoside triphosphate hydrolase
wsnp_RFL_Contig3917_4326857	CGGAAAAGCCAGGTGTGCCTCTCGTTCTTCGATGAGAAGAACAAGCACCC[A/G]GGCTGGTTCAGCAGCAAGACTGAGAGGGTTTACTGGGAACAATGGTTCAT	*TraesCS2B02G546800.1*-*TraesCS2B02G546800.2*	2B	743,745,544	743,745,644	Ubiquitin-associated domain, Ubiquitin-like domain
		*TraesCS2B02G546900.1*				Pectin methylesterase inhibitor domain superfamily, Pectinesterase inhibitor domain
BS00086051_51	CCTGGATGGTGTTTTTACCTAGCGCTGTTTGTAAGTCTTAAGTGGGTCGA[A/G]CCAGTGGTGGATCCCTGTTGCATAACTGACATTATTTATCAGGTTAGTTC	*TraesCS3A02G523700.1, TraesCS3A02G523800.1*	3A	739,147,868	739,147,978	Pentatricopeptide repeat
		*TraesCS3A02G523900.1*-*TraesCS3A02G523900.2*				Glycerol-3-phosphate dehydrogenase, NAD-dependent
Tdurum_contig12159_468	GTTTTGAACTAAAACCACGACGAGTAAATCGGAACGGAGGGAGTACATCA[T/C]TGCTATTTTACATCACCAGCTTCAGTTGTACAGACTAATTGGCTATTGGG	*TraesCS2B02G585100.1*-*TraesCS2B02G585100.2*	2B	772,053,187	772,053,297	Methyltransferase type 11, S-adenosyl-L-methionine-dependent methyltransferase
Ku_c1932_1583	TGCCGTCTTCTTTTTCTCCGTTTCCGCAGACTTAGAACCTAGACTGAGAT[T/C]GTGGCGCCTTTGCCATCCTTGTCGCGCTCTCGTTTTTAGCCTAGCCCCAT	*TraesCS1B02G354200.1*-*TraesCS1B02G354200.1*	1B	584,156,259	584,156,369	P-loop containing nucleoside triphosphate hydrolase
		*TraesCS1B02G354500.1*-*TraesCS1B02G354500.2*				Pectinesterase

Chr: Chromosome.

### Comparison with historical QTLs

**Figure 4:**
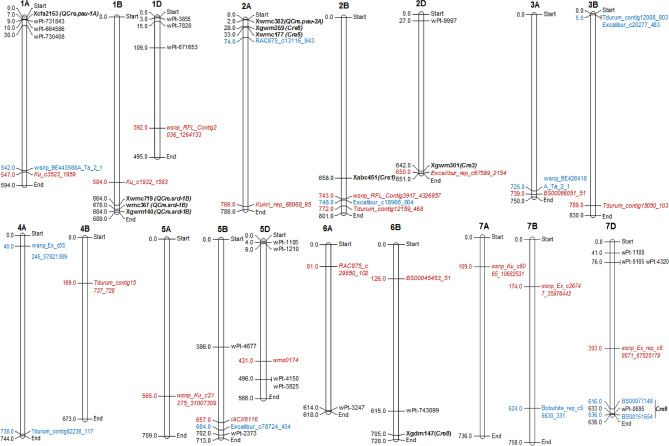
Chromosome maps showing QTLs detected in the present study and genes/QTLs/MTAs of *H. avenae* and *H. filipjevi* reported in the previous studies. Out of 19, 18 QTLs could be shown in the maps. *QCcn.ha-4A* could not be shown as suitable hits was not found. The markers associated with QTLs detected in the present study are depicted in red italicized font. Previously reported genes and QTLs for *H. avenae* are shown in black coloured bold font with associated markers shown in italicized font. Previously reported MTAs for *H. avenae* are shown in normal black font. Previously reported MTAs for *H. filipjevi* are shown in normal blue font.

The physical position of previously reported genes, QTLs and marker trait associations (MTAs) associated with resistance to *H. avenae* and *H. filipjevi* in wheat are summarized in Supplementary Tables S7, S8. The representative chromosomal maps depicting comparative presentation of all QTLs detected in the present study and previous reported QTLs/MTAs are shown in Fig. 4.

## Discussion

The ultimate goal of understanding the genetics of plant disease resistance is to uncover resistance loci and subsequently use this information to develop high yielding resistant cultivars. An understanding of the genetics governing resistance to CCN in wheat and the identification of molecular markers associated with the resistance gene(s) will facilitate the efforts of introgression resistance genes/QTLs into wheat. Although only 114 DH lines were used in the present study but dense genotyping allowed identification of 19 QTLs. Six of these 19 QTLs colocalized or nearby to previously reported *Cre* genes, QTLs or MTAs for *H. avenae* or *H. filipjevi*, while the remaining 13 QTLs were novel. Nine QTLs were detected in all three data sets. This also suggests the robustness of phenotyping and QTL analysis can be affected by several factors that include type and size of mapping, number of markers, genetic background and mapping methods^38^. As suggested in previous studies also, inconsistent QTL detection across three data sets, in the present study, might happen due to no expression or weak expression of the QTLs^39–41^.

In the present study, a highly significant QTL (*QCcn.ha-2D*), linked with SNP marker Excalibur_rep_c67599_2154 was mapped on chromosome 2D. This QTL lies at 136.0–136.40 cM interval in the distal end of long arm of chromosome 2D. The resistance in D genome against *H. avenae* was first reported in *Ae. tauschii* and synthesized allo-hexaploid wheat by Eastwood et al.^15^. Several other studies reported the presence of a resistance gene against *H. avenae* on chromosome 2D^16,42–45^. Interestingly, *QCcn.ha-2D* (detected in the present study) is co-localized with the *Cre3* resistance gene (Fig. 4), which was originally identified in *Ae. tauschii* and introduced into cultivated wheat via a synthetic hexaploid^15,44^. By constructing two genetic linkage maps of wheat chromosome 2DL, the authors were able to develop a diagnostic microsatellite marker Xgwm301 that mapped very close to the *Cre3* gene. Al-Doss et al.^46^ also confirmed the presence of *Cre3* gene on 2DL using Xgwm301 marker in another population. Eastwood et al.^16^ and Ogbonnaya et al.^19^ also developed an RFLP marker csE20-2 and a dominant PCR marker Cre3spf/r with complete linkage to *Cre3* gene. These markers were used to identify CCN resistance in wheat cultivars^47,48^.

Two QTLs identified on the distal region of long arm of 2B chromosome in the present study, namely *QCcn.ha-2B.1* and *QCcn.ha-2B.2* are linked to markers wsnp_RFL_Contig3917_4326857, 109.7–111.3 cM interval and Tdurum_contig12159_468, 87.50–130.0 cM interval, respectively. Both these QTLs are co-localized with the previously reported *Cre1* gene (Fig. 4) and impart resistance to *H. avenae* in wheat^24,49,50^. This was the first gene reported for *H. avenae* resistance in wheat. Given that both these QTLs were discovered on chromosome 2B, they are almost certainly homeoloci of *QCcn.ha-2D* as also supported by a previous study^43^.

Interestingly, we detected a QTL, *QCcn.ha-2A* (Kukri_rep_c68068_95), explaining 4.0% phenotypic variation, and located on chromosome 2A (distal end ~ 113.4 cM). This new QTL could be homeolologous to *QCcn.ha-2D*, *QCcn.ha-2B.1* and *QCcn.ha-2B.2* (Table 1; Fig. 4). This hypothesis is further supported by the detection of a significant MTA in the same region of the 2A chromosome (unpublished GWAS results). Further, epistatic interaction (additive × additive effect) was also observed (discussed later) between *QCcn.ha-2D* region and other loci on 2A (119.8 cM). However, a more detailed analysis, with additional plant material and marker enrichment for this particular chromosomal region is required to confirm this assumption. Based on the present study, it is evident that when the Indian pathotype was used for inoculation, detection of this novel QTL was possible, suggesting pathotype or race specific resistance expression.

On chromosome 1B another minor QTL linked to Ku_c1932_1583 was identified; the physical location of this QTL is near to the QTL, *QCre.srd-1B* as also reported by William et al.^51^ and Jayatilake et al.^52^. On chromosome 5B, significant QTL *QCcn.ha-5B* with LOD score ranging between 4.39 and 5.86 was identified. These QTLs co-localized with the previously identified MTA reported by Dababat et al.^53^. On chromosome 5D another minor QTL linked to wms0174 was also identified. These QTLs co-localized with the previously identified MTA reported by Mulki et al.^54^ (Table 1; Fig. 4).

In the present study, the following five novel QTLs were identified: *QCcn.ha-1D, QCcn.ha-3B, QCcn.ha-6B, QCcn.ha-7B* and *QCcn.ha-7D*; these were located on chromosomes 1D, 3B, 6B, 7B and 7D. Through association mapping studies, Dababat et al.^53^ and Mulki et al.^54^ reported the presence of MTAs for *H. avenae* resistance, with different genetic and physical positions, on chromosomes 1D, 6B and 7D in synthetic hexaploid wheat and CIMMYT advanced spring wheat lines (Table 1; Fig. 4). William et al.^51,55^ reported the resistance gene, *Cre8* and a major QTL on the long arm of chromosome 6B against *H. avenae* in the DH population of Trident/Molineux. William et al.^55^ also validated the linkage of *H. avenae* resistance QTL/gene with the RFLP marker Xcdo347-6B in another DH population of Barunga/Suneca. Jayatilke et al.^52^ improved Trident/Molineux linkage map by adding 600 markers and confirmed the *Cre8* locus near the distal region of chromosome 6BL as a major QTL.

Two minor QTLs, namely *QCcn.ha-1A* and *QCcn.ha-1B*, were also detected in the present study on chromosome 1A and 1B, respectively. Singh et al.^56^ and William et al.^51^ reported QTLs, *Qcre.pau-1A* and *QCre.srd-1B* on chromosomes 1A and 1B, respectively. In a recent study, Al-Ateeq et al.^57^ a major novel resistance locus was mapped on chromosome 1BS.

Based on the published literature, 25 putative candidate genes were identified related to biotic stress or disease resistance including genes involved in PPN resistance (for details see Supplementary Table S4). For *QCcn.ha-2A*, two candidate genes encoding Homeobox domain, Peptidase T2 and Asparaginase2 were identified. Homeobox transcription factor is involved in maintaining the feeding sites of nematode *Meloidogyne javanica* infection in tomato^58^ and also reported to be involved in rust resistance in wheat^59^. Two genes encoding Glycosyltransferase family 92 and family 8 were linked to QTLs *QCcn.ha-7D* and *QCcn.ha-1A*, respectively. This gene was reported to be involved in resistance for *M. incognita* (root-knot nematode) in tomato^60^, *H. avenae* in barley^61^ and *Fusarium* head blight (FHB) in wheat^62^. For *QCcn.ha-1A,* Galacturonosyltransferase was also found. Galacturonosyltransferase is involved in susceptibility in *Gossypium hirsutum* at the later stage of *M. incognita* infection^63^. *In-silico* expression of identified candidate genes including homeobox domain, glycosyltransferase family 92, 8 and galacturonosyltransferase showed expression in roots at different developmental stages further suggesting a possible potential role of these candidate genes in nematode resistance. However, further delimitation of the candidate genic region is required in order to identify real candidate genes underlying the main effect QTLs in the above identified genes^64^.

Inter-locus interactions i.e., gene-by-gene interactions or epistasis and gene-by-environment interactions in QTL mapping improve the efficiency of QTL discovery. However, as also suggested in the case of soybean cyst nematode (*Heterodera glycines*)^65^, phenotyping for *H. avenae* in the present study was performed in a controlled environment and hence gene-by-environment interactions were not considered in QTL analysis. Hence, in the present study, epistatic interactions along with QTLs with additive effects were addressed for QTL mapping of *H. avenae* resistance. Although few studies have focused on the effect of epistasis of QTLs for soybean cyst nematode (SCN), *H. glycines* resistance^65–68^, root-knot nematode (RKN), *M. incognita* resistance^69,70^, and no epistatic interaction study has been conducted for *H. avenae* resistance yet. In the present study, we also conducted epistatic interaction analysis to detect the interaction of main effect QTLs with other loci (Table 2; Supplementary Table S3). Epistatic interaction involving *QCcn.ha-2D* showed reduced phenotypic effect suggesting a possible application of *QCcn.ha-2D* alone in pre-breeding programme as a major gene which will likely show stable performance across different environments. Other main effect QTLs with smaller phenotypic effects showed improved phenotypic effect when they interact with other loci suggesting their possible utility, in combination, for MAS.

**Figure 5:**
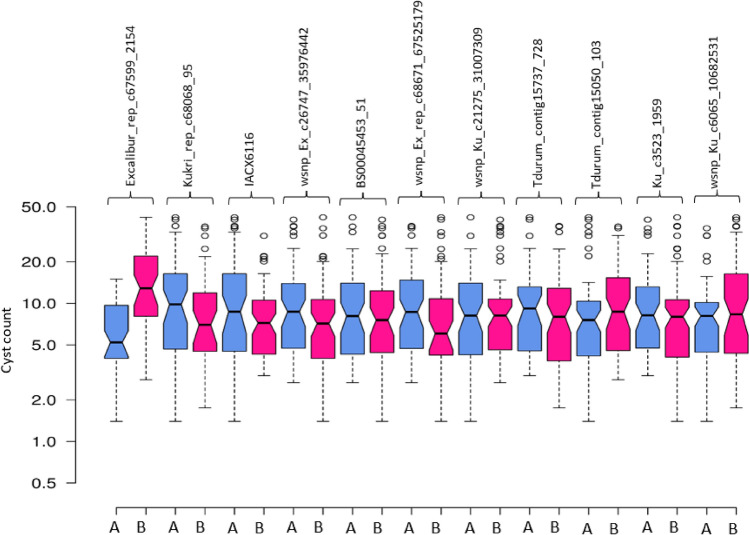
Box plots showing alleles contributing resistance or susceptibility for *Heterodera avenae*. The phenotypic value (cysts count) of 11 significant marker alleles for *H. avenae* resistance QTLs detected in Y1, Y2 and CY was selected to calculate the allelic effect. Center lines show the medians; box limits indicate the 25th and 75th percentiles; whiskers extend to 5th and 95th percentiles, outliers are represented by dots.

**Figure 6:**
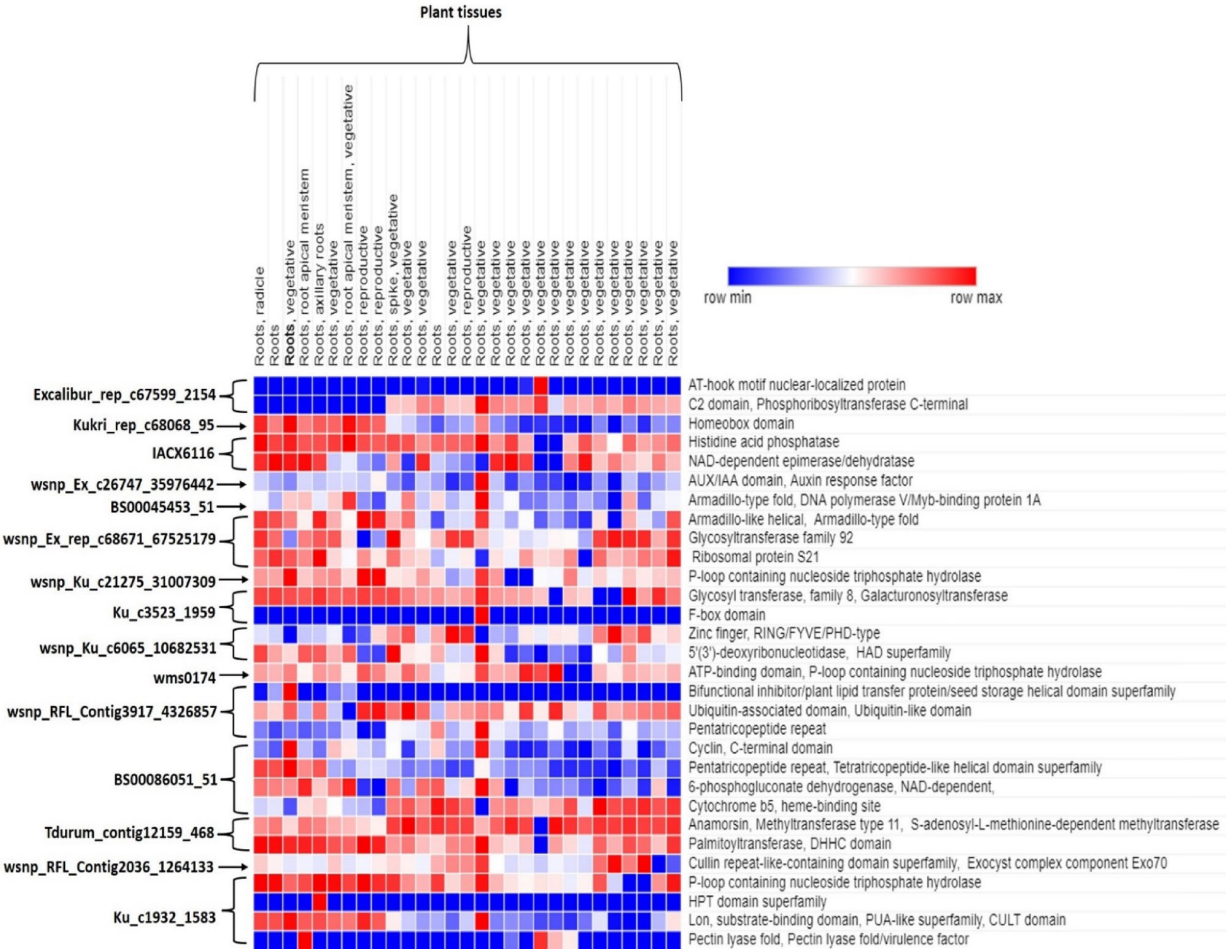
Expression profiles of candidate genes in wheat roots. Blue, white and red indicate low, medium, and high expression, respectively.

Allelic contribution may be quite effective in improving the resistance in wheat by marker-assisted breeding. Highly favorable alleles detected in the present study can be used for developing nematode resistant wheat genotypes. Alleles leading to the decrease in cyst count (Fig. 5) were considered as favorable alleles. While developing the ITMI mapping population, W7984 (M6), was used as a female parent and Opata M85 was used as a male parent^71^. W7984 (M6) and Opata M85 are originally resistant and susceptible to *H. avenae* pathotype infection, respectively. Interestingly, results of the present study showed that both parents contributed favourable alleles (Table 1; Fig. 6). For two stable QTLs (*QCcn.ha-2D* and *QCcn.ha-3B*), allele from the resistant parent led to the maximum decrease in cyst count (Fig. 5). Transfer of favourable alleles from the susceptible parent (Opata M85) suggests that expression of resistant favorable alleles remain masked in the susceptible parent and uncovered after recombination with the resistant parent.

## Conclusion

Identification and selection of resistant genotypes (DH lines here) with resistance loci and tolerance genetic loci may be useful for marker assisted backcrossing (MAB) to impart resistance against *H. avenae*. Detection of markers linked to resistance and identification of genotypes with favorable alleles for the mapped QTLs and the identified QTLs can speed up the MAB programme. The following conclusions can be drawn from the present study: except, *QCcn.ha-2D*, most of the significant main effect QTLs explained a small fraction of the observed phenotypic variation. Therefore, pyramiding these QTLs while taking into account positive/negative interaction may be the most effective strategy for increasing resistance to *H. avenae*. Eight minor QTLs (detected in all data sets) explained a combined 14% phenotypic variation. Thus, while planning a pre-breeding strategy of introducing these QTLs in pre-breeding lines, then perhaps one needs to be careful because these smaller effect QTLs may or may not be transferred. Despite having a small phenotypic effect, combining and pyramiding of these potential loci, including loci showing epistatic effects with increased phenotypic effect, for resistance into wheat breeding lines containing other sources of resistance to CCN would potentially enhance resistance level in the field^54^. Safari et al.^27^ reported that *Cre3* locus has the greatest impact on reducing the number of female cysts, followed by *Cre1* and *Cre8*. Markers associated with QTLs detected in the present study, and colocalized with *Cre1* and *Cre3*, can be used for marker assisted breeding. Development of high-throughput marker assay like KASP (competitive allele-specific PCR) followed by transfer of these QTLs to high yielding wheat lines followed by multi-location evaluation of advanced generation lines to check their agronomic performance and adaptability to diverse environments shall be the next priority. Deciphering biological function of potential candidate genes, especially those already known for their involvement in PPNs resistance/defense, is also important.

## Methods

### Plant material

In the present study, 114 lines, out of a total of 215 F_1_-derived doubled haploid lines (designated as SynOpDH) of the novel ITMI (International Triticeae Mapping Initiative) mapping population, were used^71^. This population was obtained from a cross between CIMMYT synthetic hexaploid wheat (SHW) line W-7984 (also known as M6) and the hard red spring wheat (HRSW) Opata M85^71^. M6 is a synthetic line obtained from a cross between the durum (*T*. *turgidum* L.) genotype Altar 84 and *Ae*. *tauschii* Coss., the progenitor of the bread wheat D genome^71^. Seed material of the population was originally obtained from IPK (Institute of Plant Genetics and Crop Plant Research), Gatersleben, Germany.

In the present study, the parents of the mapping population were first screened to ascertain whether this population is segregating for the resistance to *H. avenae*. For this purpose, we screened 10 plants of each parent in batches with the prevalent local *H. avenae* population from North India. Parent M6 was found to be resistant and Opata M85 was found to be susceptible. Indian wheat variety RAJ MR1 was used as a resistant check^72^ and WH147, PBW343 were used as susceptible checks^73,74^ in all the screening experiments. The DH lines were previously genotyped with the wheat 90 K chip and the map was prepared^71^. In total, 12,093 markers (11,678 SNPs and 415 SSRs markers) were used, after filtering the genotypic data, for the QTL mapping.

### Nematode inoculums preparation and plant infection test

Cysts were extracted from the nematode-infested soil by decanting and sieving method^75^. Cysts were surface sterilized with 0.5% sodium hypochlorite (NaOCl or bleach) for 10 min and rinsed six times with distilled water. These cysts were stored at 4 °C for two months. To enhance the hatching, J2 cysts were transferred to room temperature (~ 25 °C) for 24 h before plant inoculation.

The DH lines were screened for resistance against *H. avenae* under controlled environment conditions (22 °C ± 2, 16 h light, and 8 h darkness and ~ 65% relative humidity) in the growth room. For this purpose, DH lines were grown in a completely randomized design (CRD) with a minimum of five replicates in batches (over two years). The plants were irrigated with Hoagland media at a fixed interval to maintain their growth and health. The soil used was sieved and double sterilized to ensure that no other uncontrolled infection affects the plants. Seeds were pre-germinated on a wet filter paper and then transferred into 150 cm^3^ PVC (polyvinyl chloride) pipes filled with steam-sterilized soil. Three holes were made in the soil around the stem base and juveniles were inoculated near the roots using a micropipette and roots were again covered with soil. Plants were inoculated with 1000 nematodes (J2) 10 days after germination and irrigated regularly.

### Cyst extraction and categorization of infected plants

White and brown cysts were extracted from both soil and roots after 75 days of infection by decanting and sieving method. Roots containing soil samples were collected in a beaker and then filled with water. The suspension was stirred well and left for about 30 s to allow the heavy sand and soil debris to settle down. Eventually, the content was poured through 850, 250 and 150 µm sieves. This process was repeated three times to ensure all cysts were collected. Also, roots were washed gently on the upper sieve to remove cysts attached to the roots. Cysts from both roots and soil were retained on a 250 µm sieve. Roots were also examined under the microscope to confirm the removal of cysts. Cyst counting was performed for each plant under a stereomicroscope (Nikon SMZ645).

### Statistical analysis and QTL mapping

In total, 2736 data points (variety, batches or environments and replication combinations) were obtained on the screened 114 lines. All data points were manually checked for any discrepancy before analysis. Later outlier Z-test was subsequently employed. After removing the outliers from the data mixed model analysis REML (restricted maximum likelihood) implemented in ASREML-R library in R was used to obtain BLUP values of the screened DH-lines. All terms are fitted as random and the best model was selected based on the change in log-likelihood differences. Finally, the best model was fitted to compute BLUPs of the DH lines that were subsequently used for QTL mapping.

QTL analysis was conducted on three data sets, namely Year 1 (Y1), Year 2 (Y2) and combined data (CY) for the two years using interval mapping. R/QTL^76^ was used for interval mapping in two steps. Initially, scan one function and the three methods (“em”, “hk” and “imp”) implemented in R/QTL were used. All three methods detected identical QTL peaks over the chromosomes, therefore composite interval mapping (CIM) was performed using Haley-Knott regression (hk) method in R/QTL. For CIM, the number of markers as a covariate was set at three, with a window size of 5 cM. The LOD threshold value was empirically estimated with a significance level of *p* = 0.05. Further, we focused the study on the significant QTLs that surpassed the LOD value > 3.0. Besides, ICIM-EPI functions of the inclusive composite interval mapping (ICIM) program of QTL IciMapping v4.2^77^ were employed to detect possible digenic epistatic interactions between marker loci.

### Candidate gene identification and *in-silico* gene expression

For the identification of putative candidate genes and their gene ontology, the sequence of flanking or linked markers of the identified QTLs were blasted against the *T. aestivum* genome sequence information hosted at Ensembl plant release version 52 database (http://plants.ensem bl.org/Triticum_aestivum). Important genes within the interval with known functions for disease resistance were shortlisted and considered as potential candidate genes for CCN resistance. The protein products of genes present in the flanking sequence available for the marker with maximum bases (50,000 bases before and after the marker) were reported as putative genes.

The identified putative candidate genes (CGs) were subjected to *in-silico* expression analysis considering different tissue and developmental stages of wheat via the ‘Wheat Expression Browser-expVIP’ (expression Visualization and Integration Platform; http://www.wheat-expression.com)^78,79^. Further, among all tissues, roots were selected to check the expression of candidate genes at the specific developmental stage.

### Comparison of QTLs detected to historical MTAs or QTLs

The QTLs identified in the present study were also compared with previously reported genes/QTL/MTAs for *H. avenae* and *H. filipjevi.* The physical positions of all QTLs detected in the present study and previously reported QTL/MTAs were located through Ensembl Plants [version 52https://plants.ensembl.org/Triticum_aestivum/Info/Index]. Results were compared based on the physical position of the particular marker on a particular chromosome. The representative chromosomal maps of all QTLs detected in the present study and previously reported QTLs/MTAs on an individual chromosome were also prepared using Map chart software.

## Supplementary Information

Supplementary Information 1.

Supplementary Information 2.
