# Supplementary material for: QTL mapping for resistance against cereal cyst nematode (Heterodera avenae Woll.) in wheat (Triticum aestivum L.)
Source: Sci Rep. 2022 Jun 10;12:9586. doi: 10.1038/s41598-022-12988-7 (PMC9187758; doi:10.1038/s41598-022-12988-7)
Supplement: Supplementary file 1 — Supplementary Information 1. [file 41598_2022_12988_MOESM1_ESM.docx]

**QTL Mapping for Resistance against Cereal Cyst Nematode (*Heterodera avenae* Woll.) in Wheat (*Triticum aestivum* L.)**

Saksham Pundir^1,2^, Rajiv Sharma^3^, Deepak Kumar^1,2^, Vikas Kumar Singh^1^, Deepti Chaturvedi^1^, Rambir Singh Kanwar^4^, Marion S Röder^5^, Andreas Börner^5^, Martin W Ganal^6^, Pushpendra Kumar Gupta^1^, Shailendra Sharma^1^, Shiveta Sharma^1^*

1. Department of Genetics and Plant Breeding, Chaudhary Charan Singh University (CCSU), Meerut 250 004, Uttar Pradesh, India.
2. Department of Botany, Chaudhary Charan Singh University (CCSU), Meerut 250 004, Uttar Pradesh, India.
3. Scotland's Rural College (SRUC), Peter Wilson Building, West Mains Road, EdinburghEH9 3JG United Kingdom
4. Department of Nematology, Chaudhary Charan Singh Haryana Agricultural University (CCSHAU), Hisar 125 004, Haryana, India.
5. Leibniz Institute of Plant Genetics and Crop Plant Research (IPK), Corrensstrasse 3, 06466 Seeland, OT Gatersleben, Germany.
6. Trait Genetics GmbH, Am Schwabeplan 1b, 06466 Seeland, OT Gatersleben, Germany.

*Corresponding author:

Shiveta Sharma,

Email: s2sbhu@gmail.com

Department of Genetics and Plant Breeding,

Chaudhary Charan Singh University,

Meerut-250004 (U.P), India

Email: s2sbhu@gmail.com


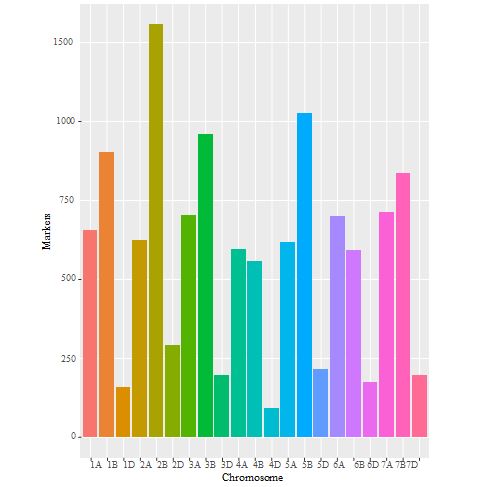


1500

1000

750

Frequency of the Markers

500

250

0

1A 1B 1D 2A 2B 2D 3A 3B 3D 4A 4B 4D 5A 5B 5D 6A 6B 6D 7A 7B 7D

**Supplementary Figure S1.** Distribution of markers across all the chromosomes of wheat genome.


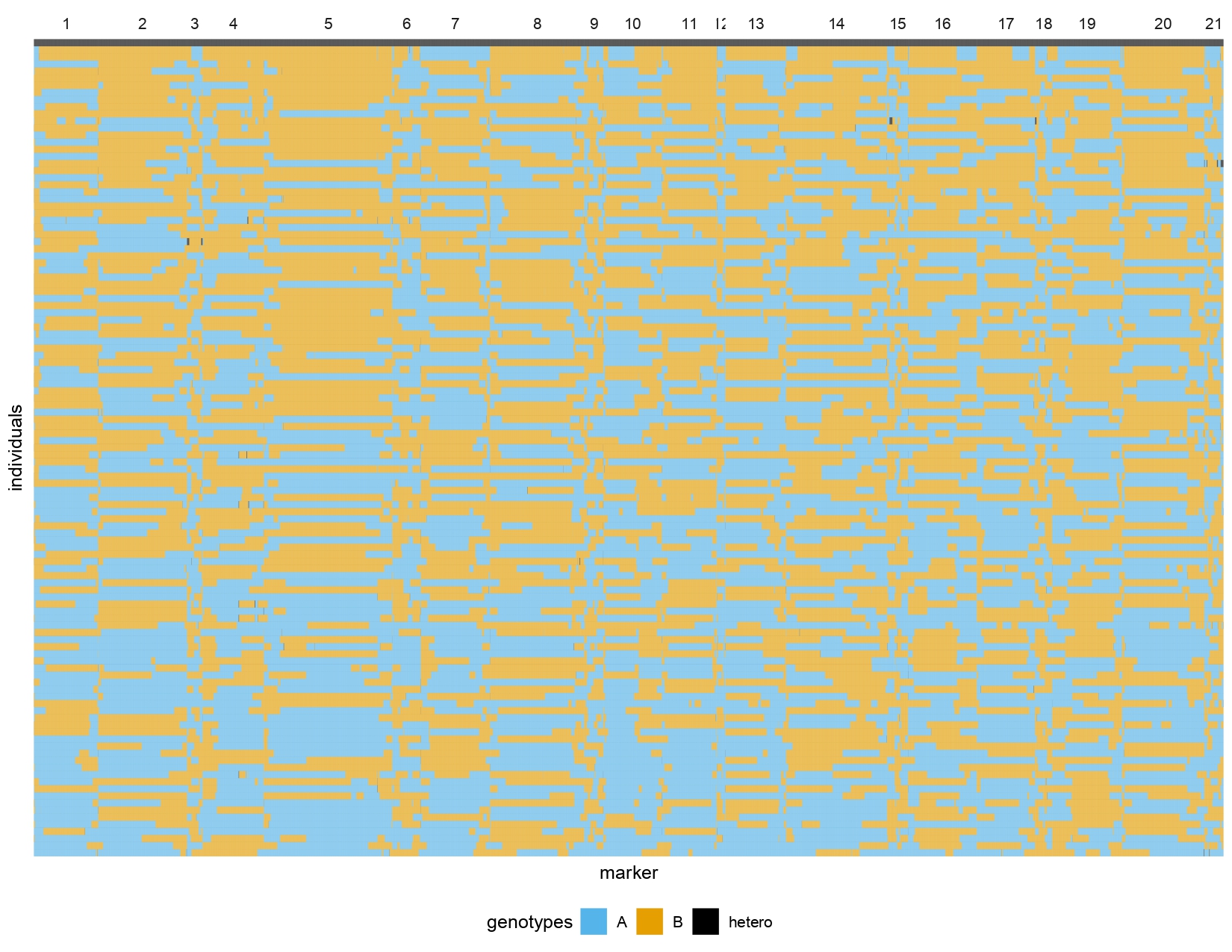


DH Lines

**Supplementary Figure S2.** Graphical genotype data of the doubled haploid (DH) lines. Displaying 21 wheat chromosomes on the Y-axis and the different DH lines on the X-axis. Blue color= M6 and Yellow= Opata genomic regions.


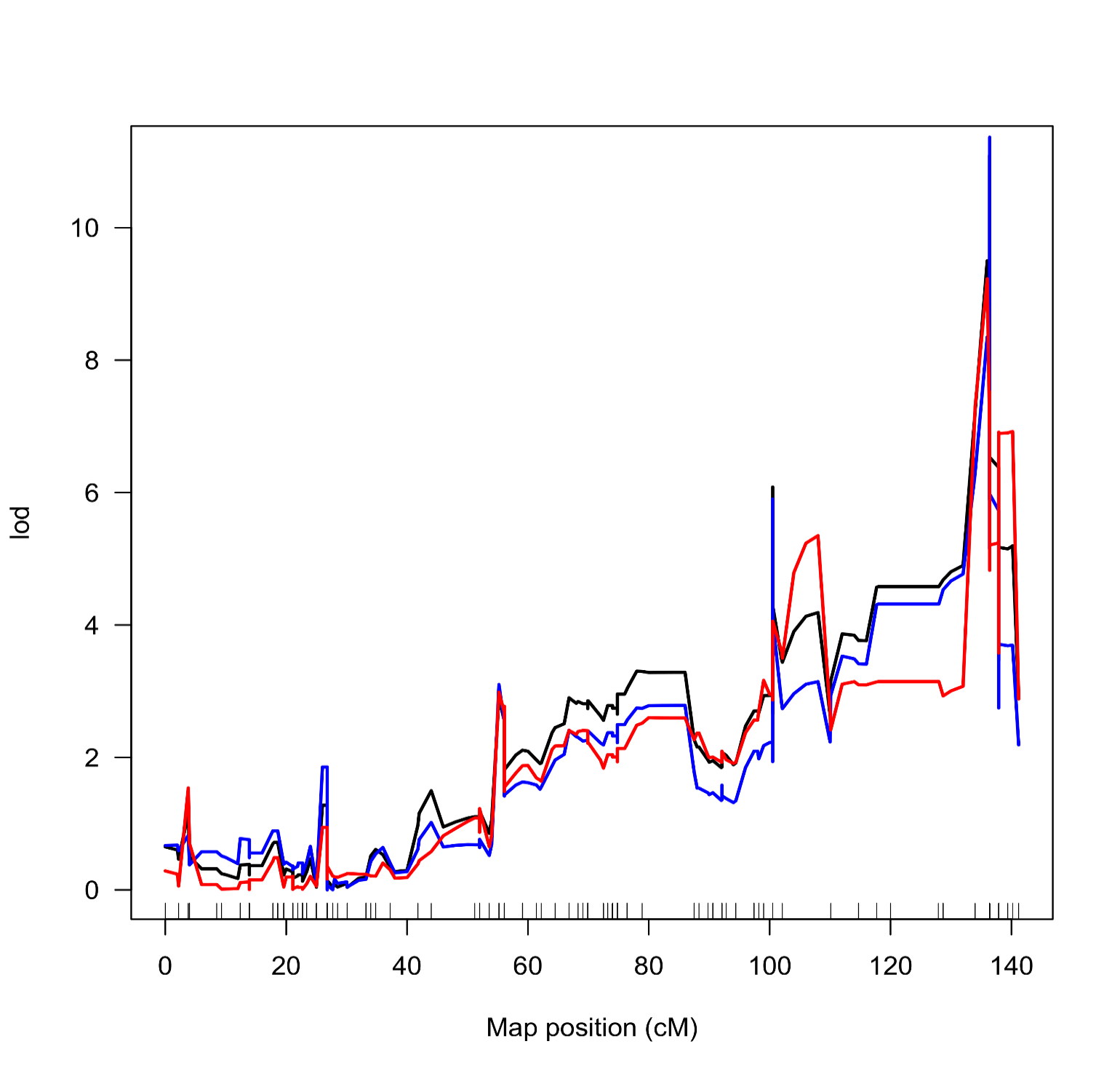


**Supplementary Figure S3.** Major QTL profile detected on the long-arm of chromosome 2D using CIM. Blue= Year 1 (Y1), Red= Year 2 (Y2) and Black= Combined data (CY).


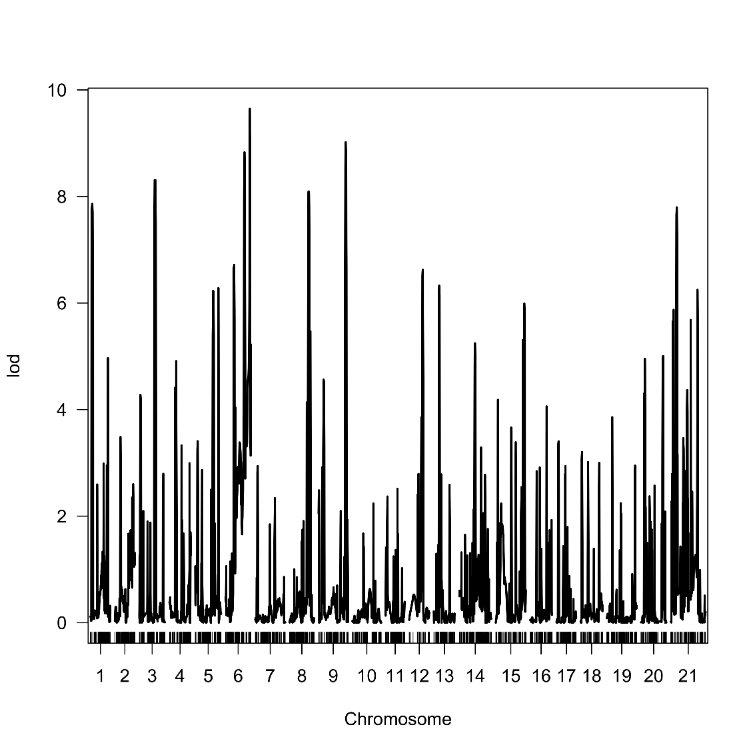

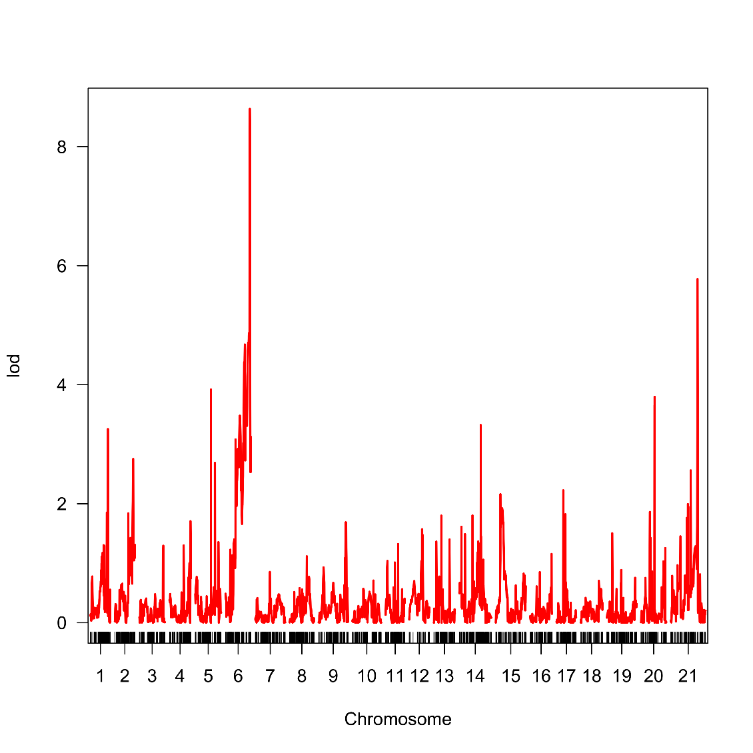

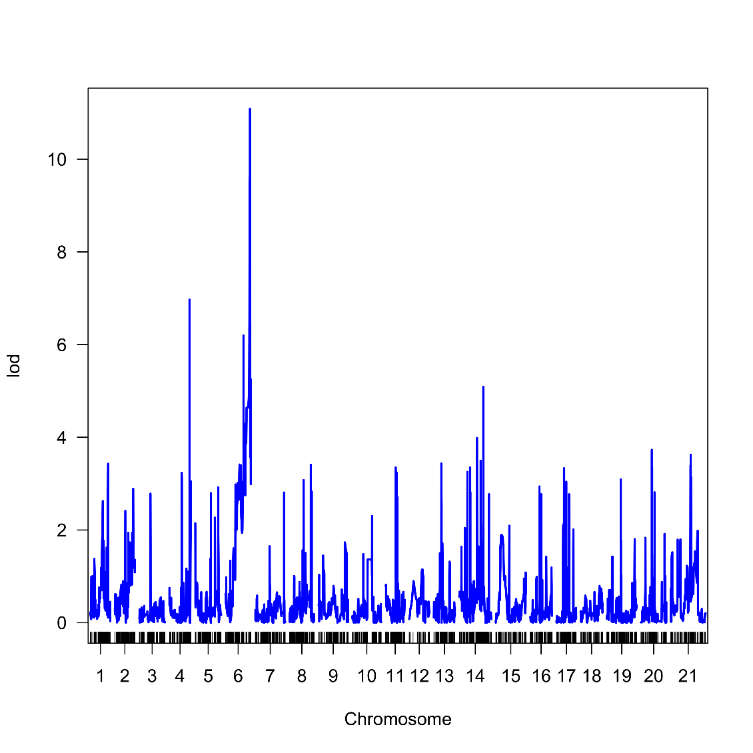

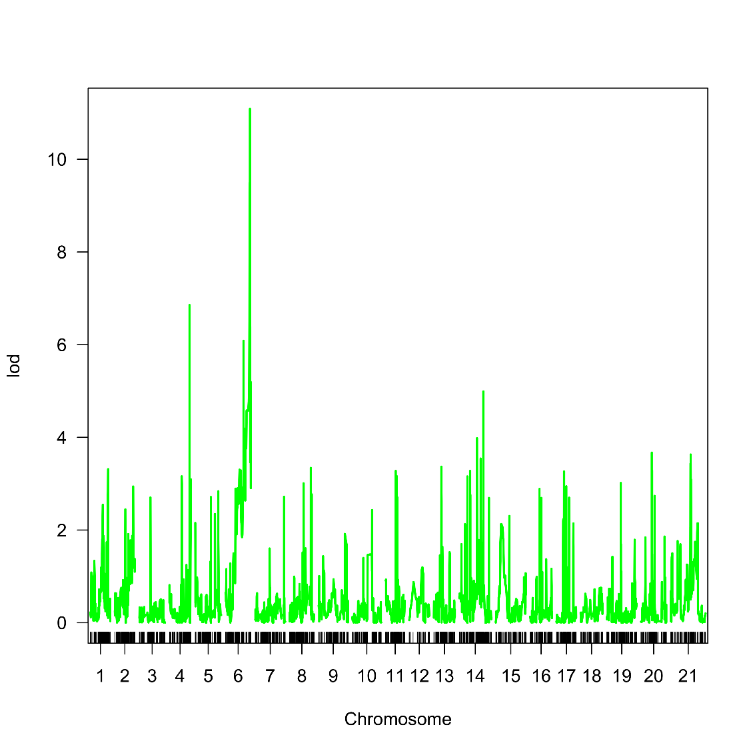


Chromosomes

Chromosomes

Chromosomes

Chromosomes

LOD

LOD

LOD

LOD

**(a)**

**(b)**

**(d)**

**(c)**

**Supplementary Figure S4.** Genome-wide QTL analysis using combined data (CY). QTL analysis: Black= “em” method (a), Blue= “hk” method (b), Red= “imp” (c) and Green= “cim” method (d).


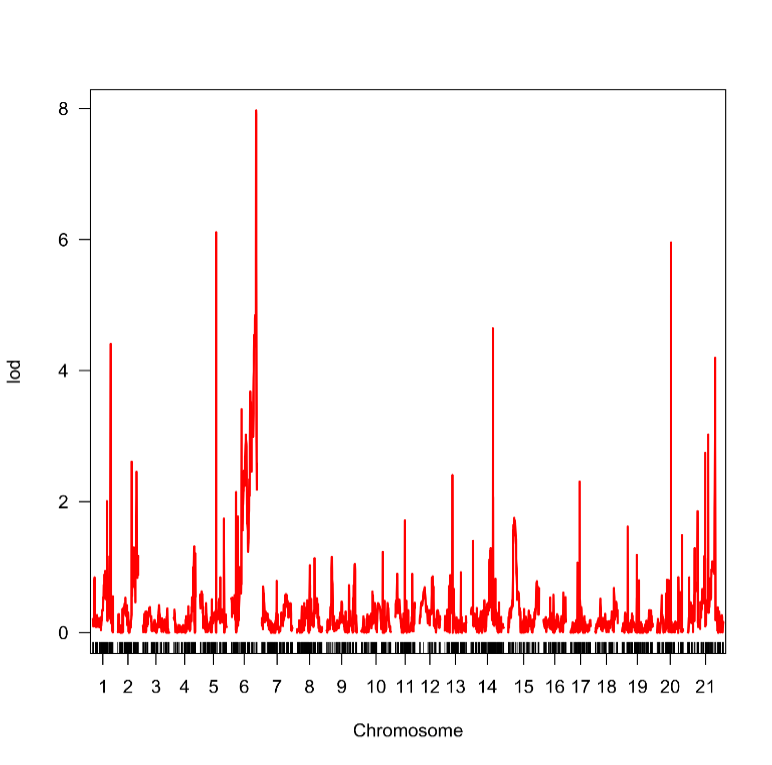

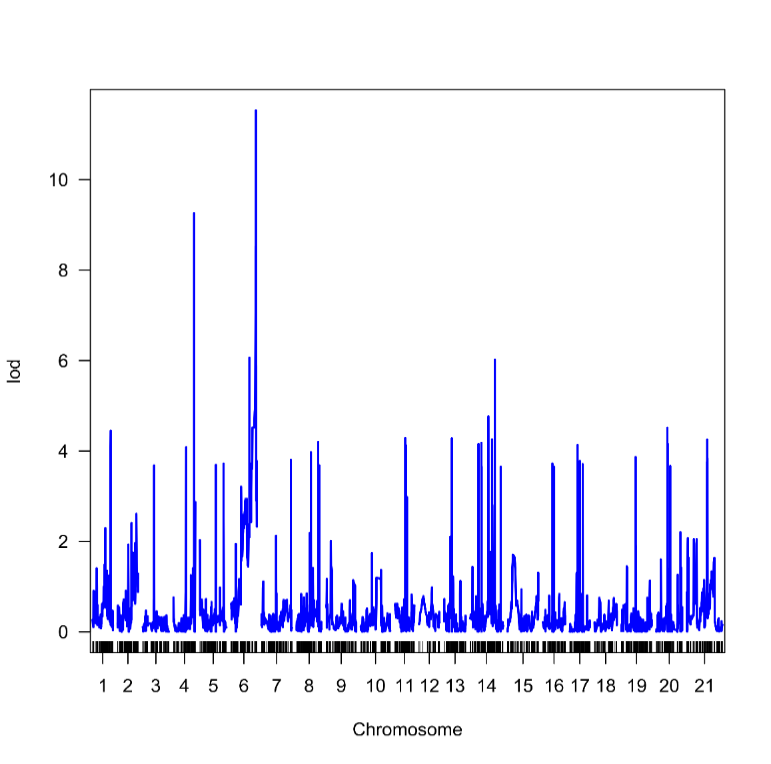

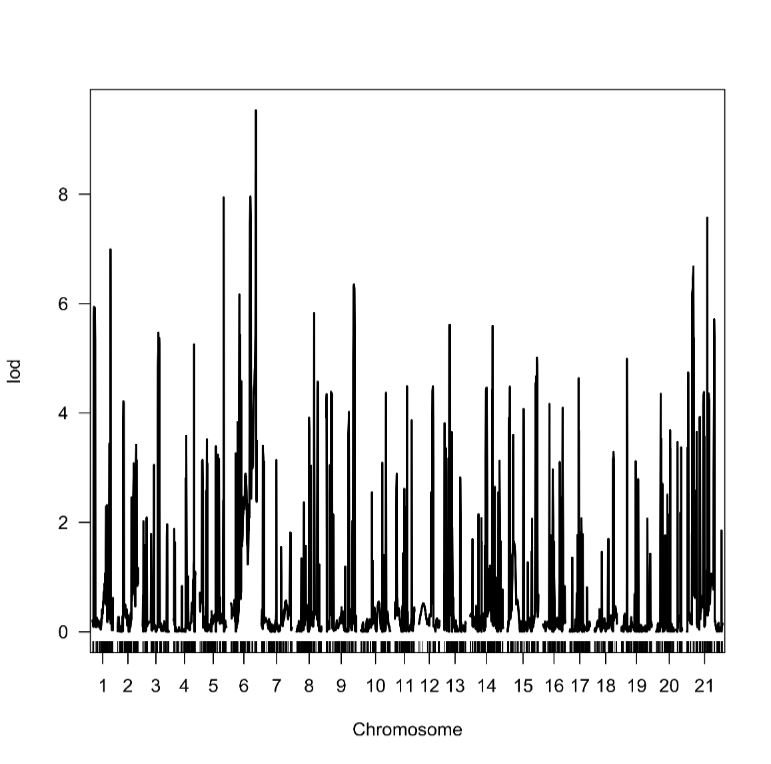

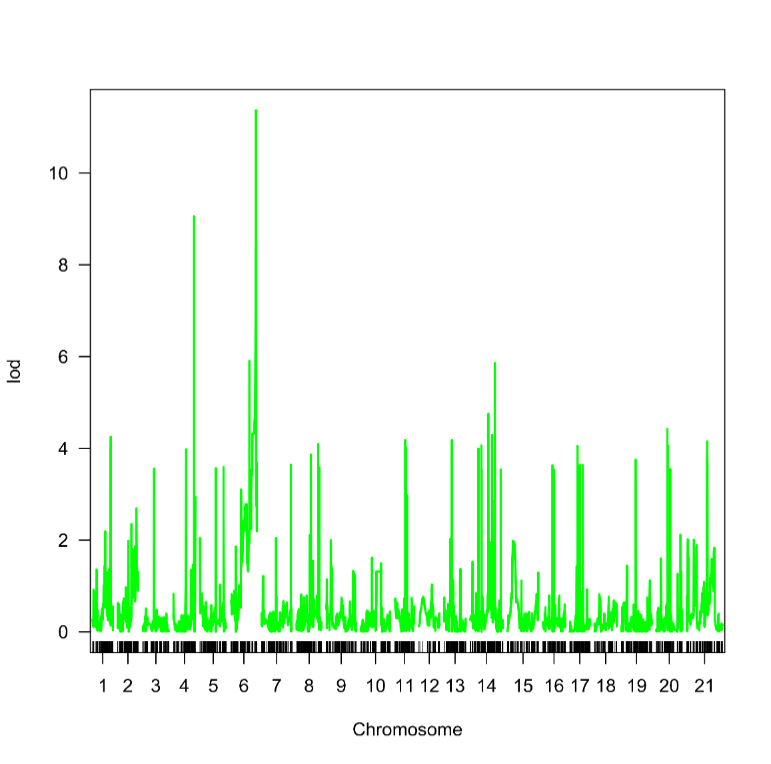


Chromosomes

Chromosomes

Chromosomes

Chromosomes

LOD

LOD

LOD

**(a)**

**(b)**

**(d)**

**(c)**

**Supplementary Figure S5.** Genome-wide QTL analysis using year 1 data (Y1). QTL analysis: Black= “em” method (a), Blue= “hk” method (b), Red= “imp” (c) and Green= “cim” method (d).


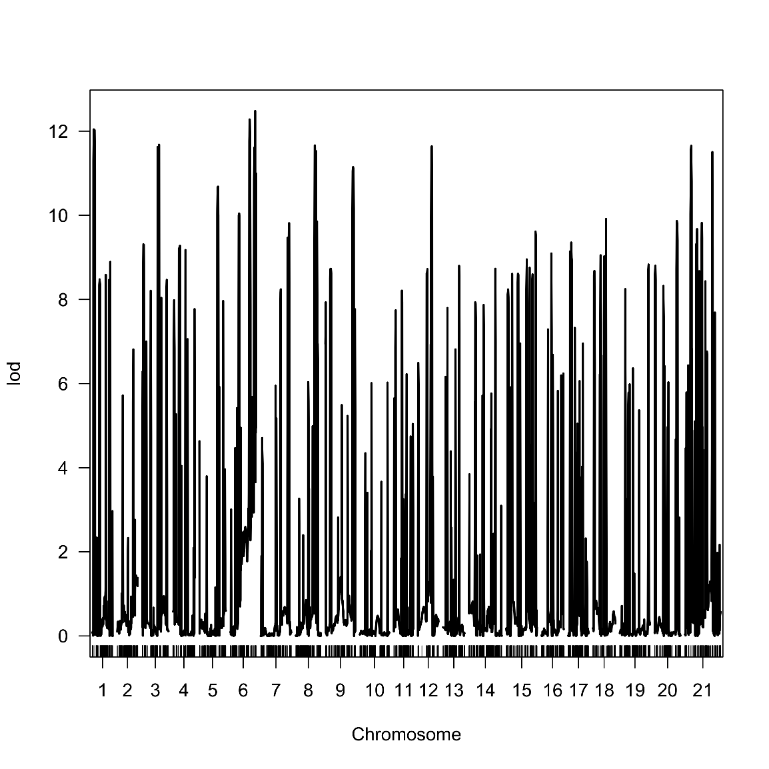

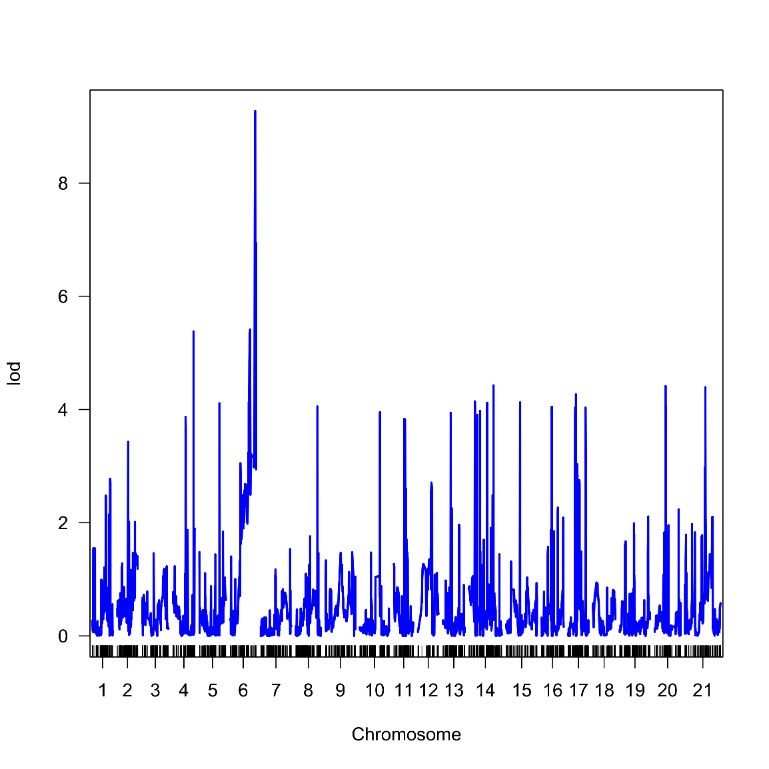

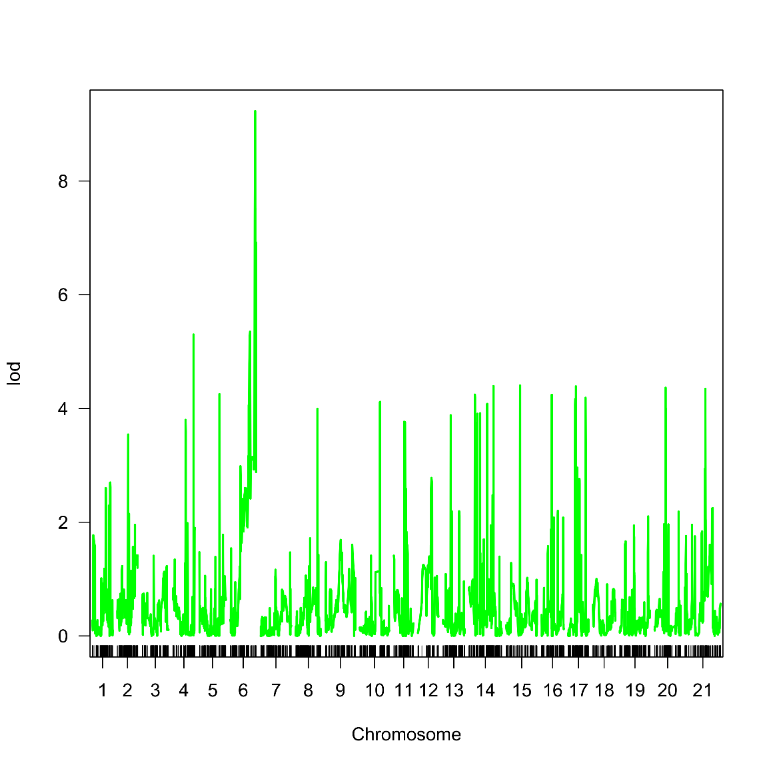

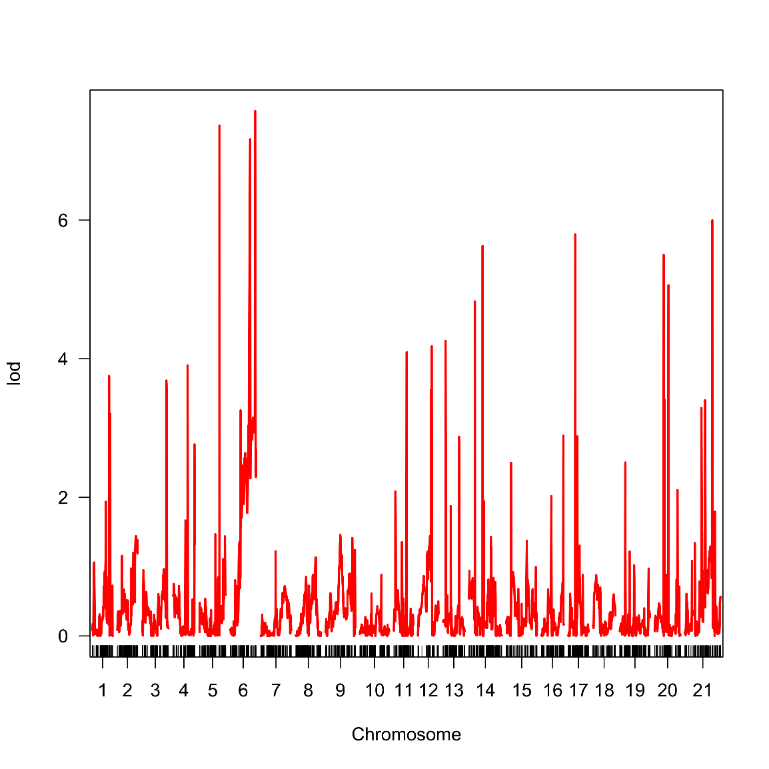


Chromosomes

Chromosomes

Chromosomes

LOD

LOD

LOD

LOD

Chromosomes

**(a)**

**(b)**

**(d)**

**(c)**

**Supplementary Figure S6.** Genome-wide QTL analysis using year 2 data (Y2). QTL analysis: Black= “em” method (a), Blue= “hk” method (b), Red= “imp” (c) and Green= “cim” method (d).
